# P-793. Antimicrobial Resistance of Uropathogens in Diabetic Patients

**DOI:** 10.1093/ofid/ofaf695.1003

**Published:** 2026-01-11

**Authors:** Yazeed Y Alajlouni, Anas Abu-humaidan, Omar Hamdan, Amin Alajlouni, Batool Basyouni, Ola Qasem, Abdallah Riyalat, Ruba Hiasat, Anmar Magahrbeh

**Affiliations:** University of Jordan, school of medicine, amman, 'Amman, Jordan; University of Jordan, school of medicine, amman, 'Amman, Jordan; University of Jordan, school of medicine, amman, 'Amman, Jordan; School of Medicine, Al-Balqa' Applied University, Amman, 'Amman, Jordan; School of Medicine, Al-Balqa' Applied University, Amman, 'Amman, Jordan; School of Medicine, Al-Balqa' Applied University, Amman, 'Amman, Jordan; School of Medicine, Al-Balqa' Applied University, Amman, 'Amman, Jordan; School of Medicine, Al-Balqa' Applied University, Amman, 'Amman, Jordan; School of Medicine, Al-Balqa' Applied University, Amman, 'Amman, Jordan

## Abstract

**Background:**

Patients with Diabetes mellitus (DM) have higher rates of urinary tract infections (UTIs). This study evaluated whether uropathogens isolated from DM patients exhibit greater antimicrobial resistance (AMR).

**Table 1.**
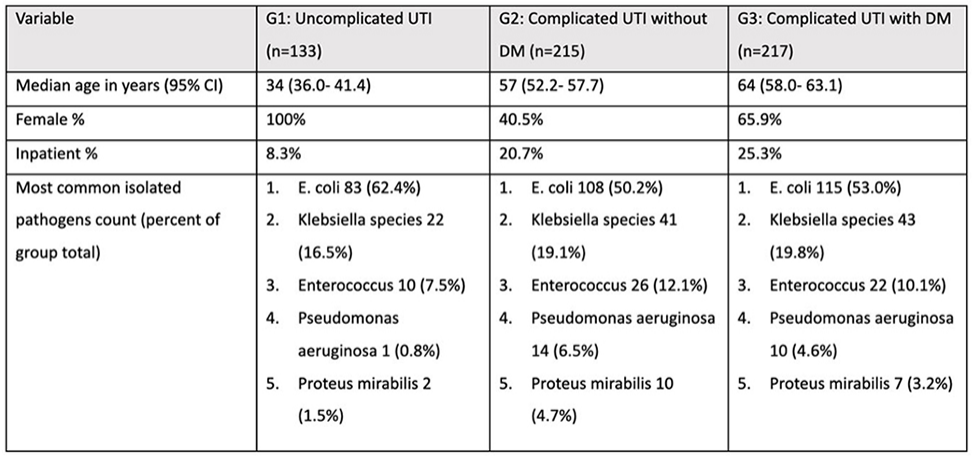
Demographics of patient groups and the isolated uropathogens

**Figure 1.**
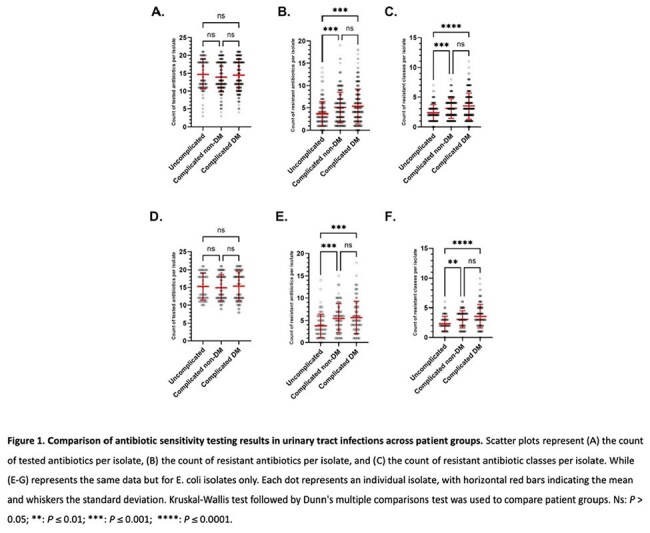
Comparison of antibiotic sensitivity testing results in urinary tract infections across patient groups. Scatter plots represent (A) the count of tested antibiotics per isolate, (B) the count of resistant antibiotics per isolate, and (C) the count of resistant antibiotic classes per isolate. While (E-G) represents the same data but for E. coli isolates only. Each dot represents an individual isolate, with horizontal red bars indicating the mean and whiskers the standard deviation. Kruskal-Wallis test followed by Dunn's multiple comparisons test was used to compare patient groups. Ns: P > 0.05; **: P ≤ 0.01; ***: P ≤ 0.001; ****: P ≤ 0.0001.

**Methods:**

A cross-sectional multicenter study included adults with UTI symptoms and a positive urine culture. Patients were categorized into uncomplicated UTI (G1, n=133), complicated UTI without (G2, n=215) or with DM (G3, n=217). Patient data, microbiology results, and associations between DM, antibiotic resistance, and bacterial isolates were analyzed using Kruskal-Wallis followed by Dunn's multiple comparisons, or Fisher's exact test as appropriate.

**Figure 2.**
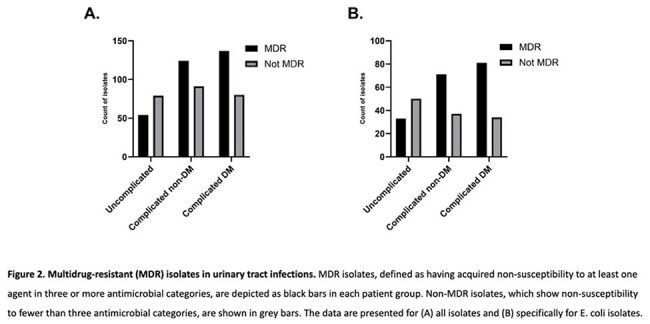
Multidrug-resistant (MDR) isolates in urinary tract infections. MDR isolates, defined as having acquired non-susceptibility to at least one agent in three or more antimicrobial categories, are depicted as black bars in each patient group. Non-MDR isolates, which show non-susceptibility to fewer than three antimicrobial categories, are shown in grey bars. The data are presented for (A) all isolates and (B) specifically for E. coli isolates.

**Table 2.**
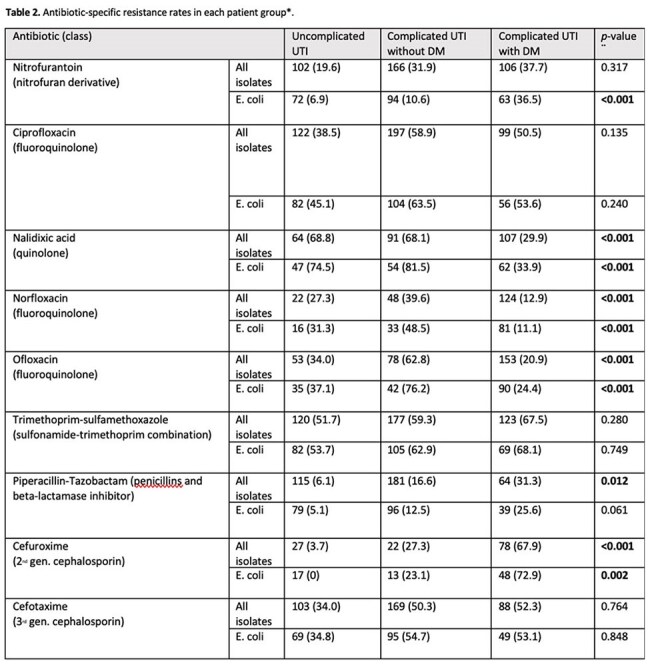
Antibiotic-specific resistance rates in each patient group.

**Results:**

The median age in years (95% CI) was as follows: [G1] 34 (36.0- 41.4), [G2] 57 (52.2- 57.7), [G3] 64 (58.0- 63.1). When considering all patients, E. coli was the most frequently isolated uropathogen (54.2%), followed by Klebsiella species (18.8%). However, the complicated UTI groups G2 and G3 had a higher percentage of non- E. coli uropathogens compared to the uncomplicated UTI group (G1), since they formed 37.6 % of G1 isolates, 49.8% of G2, and 47.0% of G3 (Table 1).

For E. coli isolates specifically, the number of antibiotics to which an isolate was resistant was significantly lower in G1 compared to G2 and G3, with a median of 3, 5, and 4 respectively (P value < 0.0001). No significant differences between G2 and G3 were found. The percent of multidrug resistant (MDR) isolates in G1, G2, and G3 was 39.8%, 65.7%, and 70.4%, respectively (Fisher's exact test P value < 0.0001) (Figure 1 and 2). Fisher's exact test revealed significant differences in the resistance to specific antibiotics between G2 and G3. For example, Resistance to Nitrofurantoin in E. coli, was notably higher in the G3 (36.5%) compared to G2 (10.6%) (p-value < 0.001), similarly, resistance to piperacillin/tazobactam and cefuroxime was significantly higher in G3 compared to G2 (Table 2). Among the 3 groups, the lowest resistance was to the carbapenems ertapenem (0%, 1.1%, and 5.1%, respectively), and imipenem (0%, 3.0%, and 2.4%, respectively).

**Conclusion:**

These findings suggest that the impact of DM on AMR is antibiotic-specific. Stratifying patients by risk factors like DM could improve empiric therapy outcomes and contribute to reducing AMR.

**Disclosures:**

All Authors: No reported disclosures

